# The Influence of Comorbidities, General Health Status, and Self-Care Self-Efficacy on COVID-19 Symptoms During the Omicron Wave

**DOI:** 10.7759/cureus.49176

**Published:** 2023-11-21

**Authors:** Mini M Jose, Juan Feng, Hoang T Nguyen, Cheryl Juneau, Bushra M Manakatt, Jennifer Barnett, Jennifer L Jones, Mukaila Raji

**Affiliations:** 1 School of Nursing, University of Texas Medical Branch, Galveston, USA; 2 Department of Internal Medicine, Division of Geriatrics & Palliative Medicine, University of Texas Medical Branch, Galveston, USA; 3 Department of Preventive Medicine and Population Health, University of Texas Medical Branch, Galveston, USA

**Keywords:** depression and covid-19, mental health and covid-19, adults with covid-19, covid-19 symptom rating scale, covid-19 outpatient care, charlson comorbidity index, self-care self-efficacy scale, sf-12, omicron, the covid-19 symptoms severity

## Abstract

Background

The emergence of the less virulent COVID-19 strains such as Omicron and its subvariants shifted the paradigm of COVID-19 treatment from inpatient treatment to regular outpatient care. The individual health determinants affecting COVID-19 disease severity among vulnerable adults treated in outpatient settings are an under-researched area.

Methods

This study conducted in an outpatient COVID-19 antibody infusion center employed a cross-sectional survey design to explore the impact of comorbidities, general health status, and self-care self-efficacy on COVID-19 symptom severity. We recruited 120 COVID-19-positive participants over 40 years of age, of which 117 completed the study with 87 providing complete data. After the screening and consenting process, the participants completed the following surveys in a secure REDCap survey software (Vanderbilt University, Nashville, USA) on an iPad (Apple Inc., Cupertino, USA): 1) sociodemographic questionnaire, 2) Charlson Comorbidity Index (CCI) to capture comorbidities, 3) Medical Outcomes Study Short-Form (SF-12) to assess general health including physical (PCS) and mental (MCS) health subscales, 4) Self-Care Self-Efficacy Scale (SCSES) to measure self-care self-efficacy, and 5) the COVID-19 Symptom rating scale (COVID-19 SRS). Statistical analysis used were Chi-square and Pearson correlations.

Results

As evidenced by CCI, the top five comorbidities were hypertension (42%), diabetes mellitus (31%), pulmonary disease (19%), depression (14%), and solid tumors (11%). Age was statistically significantly correlated to comorbidity burden (p<0.0001). Severe COVID-19 symptoms reported were fatigue, myalgia, cough, runny nose, and sore throat. The general health status measure (SF-12) subscales showed that the patient’s mental component summary (MCS) was more statistically significant to COVID-19 symptom severity than the physical component summary (PCS). The MCS demonstrated a statistically significant correlation with fatigue and myalgia (p<0.0001), headache and breathing difficulties (p<0.001), nausea/vomiting (p<0.01), and abdominal pain/diarrhea (p<0.05). The PCS showed a lesser statistically significant correlation with fatigue, myalgia, headaches (p<0.01), fever/chills, cough, congestion/runny nose, night sweats, breathing difficulties, nausea/vomiting, and abdominal pain/diarrhea (p<0.05). Interestingly, the ‘loss of smell’ which is the hallmark symptom of COVID-19 was the only symptom that showed a statically significant correlation with the Charlson Comorbidity Index (p<0.05), and it did not show any association with either mental (SF-12 MCS) or physical (SF-12 PCS) health status. The SF-12 MCS also showed a statistically significant correlation with a diagnosis of depression (p< 0.01), validating it as a true measure of mental health among vulnerable adults. The SCSES was not correlated with any of the COVID-19 symptoms.

Conclusions

The patient’s general health status, especially mental health was more statistically significant to COVID-19 symptoms. The COVID-19 hallmark symptom of ‘loss of smell’ was the only symptom that showed statistical significance with comorbidities. Within the limitations of a cross-sectional survey design and convenient sampling methods, this study calls to tailor general health status, especially mental health, and cumulative comorbidity burden to risk assessment/risk stratification of COVID-19 care.

## Introduction

The first two waves of the coronavirus disease 2019 (COVID-19) pandemic caused by highly virulent SARS-CoV-2 strains resulted in an unprecedented number of hospitalizations and more than a million deaths within the United States (https://covid.cdc.gov/covid-data-tracker/#datatracker-home). Most deaths were reported within the vulnerable population of older adults with pre-existing comorbidities. Multi-pronged infection control efforts turned the corner on SARS-COV-2 virulence and a less virulent ‘Omicron variant’ emerged causing comparatively milder COVID-19 infections. The Omicron wave was characterized by milder infections and fewer deaths, even within some of the high-risk populations [1]. Still, pre-existing co-morbidities complicated recovery from COVID-19, and the cumulative burden of multiple comorbidities was associated with poor prognosis.

Along with noticeably milder symptoms, widespread vaccination, and availability of outpatient COVID-19 treatment options, prompted a paradigm shift in COVID-19 care [2] moving it from inpatient care to outpatient treatment within the ambulatory care settings [3]. However, isolation and quarantine within the community setting are challenging for older adults with vulnerabilities. A patient’s self-care self-efficacy, general health status, and the availability of tangible social support are health determinants in the fight against COVID-19 illness in community settings. The lack of research to comprehensively understand a person’s risk factors influencing recovery from COVID-19 infection while being treated in outpatient settings makes COVID-19 treatment a guesswork for clinicians. The COVID-19 pandemic will remain a public health threat in the near future, and it is imperative to devise evidence-based strategies within the community to protect older adults with comorbidities from complications of COVID-19 [4]. The objective of this study was to assess the influence of self-care efficacy, general health status, and comorbidity burden on the COVID-19 symptom severity among adults over 40 years of age who tested positive for COVID-19 during the Omicron outbreak within the United States.

Background and significance

The third wave of COVID-19 infection with predominantly less severe ‘Omicron’ virus and its later variants, presented a unique opportunity to treat vulnerable adult patients in the outpatient setting. Though the ‘Omicron’ variant produced relatively milder symptoms, its differential impact among vulnerable populations such as older adults with comorbidities was not adequately documented in the research literature and there are challenges in improving the quality of COVID-19 care in outpatient settings. For example, The United States Centers for Disease Control (CDC) provides a COVID-19 symptom checklist to diagnose COVID-19 symptoms, but this tool does not measure symptom severity (https://www.cdc.gov/coronavirus/2019-ncov/symptoms-testing/symptoms.html). Hence, there is a need for the adaptation of the COVID-19 symptom checklist as a COVID-19 Symptom Rating Scale (COVID-SRS) as used in this study to capture the variability of symptom severity among cognitively intact COVID-19-positive patients, which can be especially useful for monitoring the symptoms in ‘long COVID’.

Rigorous studies conducted in multiple countries established that COVID-19 patients with pre-existing comorbidities had a higher likelihood of death, especially those with advanced age [5]. Multiple meta-analyses revealed that advanced age, comorbidities, and abnormal inflammatory biomarkers were predictive of poor outcomes [6]. In past studies, cardiometabolic diseases including hypertension, diabetes, cardiovascular disorders [7], and chronic kidney disease were found to be risk factors for severe COVID-19 infection [8]. However, most of those studies were conducted among patients with severe infection admitted to inpatient settings, and the risk factors were constructed based on clinical and laboratory observations, rather than a comprehensive assessment inclusive of psychosocial elements [9]. Employing clinical observations alone might result in a possible bias towards over-emphasis on physical health and exclusion of psychosocial elements from the list of relevant risk factors, which are especially important in outpatient care. Research is imperative to integrate reliable and user-friendly tools that can be used along with clinical indicators to augment the precision of risk assessment of older adults with COVID-19 receiving outpatient care while in community-based isolation. For example, recent studies found that the updated Charlson Comorbidity Index (uCCI) listing physical conditions alone is reliable in predicting the impact of comorbidities in recovery from COVID-19 [10], though the modified version of CCI (2014) including mental health conditions such as depression is yet to be tested [11,12]. Conventionally the CCI has been used as a predictor of mortality. However, this study conceives that CCI has utility as a predictor of COVID-19 symptoms and can be used as a proxy measure for function and well-being in the context of COVID-19 illness.

Given the nascent state of multiple COVID-19 treatment options, precise risk assessment is the first step to significantly reduce the number of fatalities from COVID-19 in an outpatient setting [13,14]. Rigorous research is imperative to guide comprehensive risk assessment (that covers biopsychosocial and spiritual factors) and potential risk stratification of COVID-19 patients, which cannot be merely based on comorbidities and age alone, as in the commonly used CCI. Apart from comorbidities, the patient’s general health status, self-care ability, available social support, and of critical importance-mental health status, are some of the important determinants of health, function, and quality of life to be considered while planning care in outpatient settings. A patient’s general health, especially mental health, is crucial in the defense against COVID-19 infection and its complications. Though age and comorbidities influence it, general health status is multifactorial with physical, mental, social, and spiritual components contributing to it. The Medical Outcomes Survey Short Form (SF-12) is a comprehensive, reliable, and valid tool to assess a person's general health status including physical and mental health aspects. The SF-12 is a short reliable, and valid measure to use in an outpatient setting because this includes two subscales: mental component summary (MCS) and physical component summary (PCS). Alongside the clinical indicators of the COVID-19 disease severity, using SF-12 can potentially help healthcare providers with precise identification of the health status of at-risk individuals and devise a better plan of care [15].

Pandemics are often self-limiting viral illnesses, and robust self-care can facilitate recovery from self-limiting viral illnesses like COVID-19 [16,17]. Self-care in isolation is the norm after the COVID-19 diagnosis unless there are compelling reasons for hospitalization. It is essential to screen for the barriers to successful self-care management in isolation to reduce the mortality rate from COVID-19, especially among the high-risk group of older adults with specific comorbidities. Internationally, self-care measures have been developed to screen for the general population's self-care readiness to prevent COVID-19 infection [18,19]. For example, research using a telephone survey of COVID-19 severity scores based on risk factors and symptoms of COVID helped improve community-based treatment and free up acute-care beds during the COVID-19 pandemic in South Korea [20]. The putative impact of various risk factors on patient recovery cannot be measured without a valid and reliable tool measuring the combination of risk factors [21,22]. Self-care ability is a factor to consider when patients are entrusted with their care in the community setting, more so when they are in isolation due to a highly communicable disease like COVID-19. Self-Care Self-Efficacy Survey (SCSES) is newly validated as a self-report measure for self-care self-efficacy among adults with chronic illness. Since this is a measure to assess the patient’s confidence in handling self-care, the SCSES seems to be apt to determine the vulnerable adult’s confidence to care for themselves in COVID-19-related isolation.

In summary, the outpatient management of at-risk adults with COVID-19 calls for structured risk assessment comprehensively. Along with pre-existing co-morbidities, lack of self-care self-efficacy, and poor general health status (including mental, psychological, and social factors) as important determinants of COVID-related morbidity and mortality have not been systematically assessed using standardized instruments in a population with COVID-19 disease. There is a need for rigorous research to develop a standardized approach to outpatient COVID-19 treatment of at-risk individuals, giving due consideration to the above-mentioned individual health determinants. Such understanding can guide the development of clinical practice guidelines for optimal COVID care and inform public health policy on COVID risk mitigation measures and vaccination, especially among high-risk and underserved racial/ethnic minorities [23].

## Materials and methods

This study aimed to evaluate the role of selected sociodemographic risk factors in contributing to the severity of COVID-19 symptoms. We employed a cross-sectional survey study design to assess the role of selected individual health determinants in the incidence of symptomatic COVID-19 disease with a sample of 120 patients presenting for COVID-19 treatment at the “REGEN-COV” monoclonal antibody treatment centers. The objectives of the study were to determine 1) the correlation between the comorbidity burden as measured by CCI and the severity of COVID-19 symptoms, 2) the influence of self-care self-efficacy on COVID-19 symptoms, and 3) the association of general physical and mental health status with the severity of COVID-19 symptoms.

We recruited cognitively intact and hemodynamically stable COVID-19-positive adults aged 40 years and above for this study. The potential participants who met the eligibility criteria were screened with the Short Portable Mental Status Questionnaire (SPMSQ) to screen for cognitive impairment. Once the potential participant cleared the screening process, we electronically collected self-reported data using standardized questionnaires presented in a preordered sequence using REDCap survey software (Vanderbild University, Nashville, USA) [24,25]. The participants completed surveys using an iPad (Apple Inc., Cupertino, USA) connected to the secure network. The surveys were available in English and Spanish languages and were organized in the following order: 1) a brief sociodemographic questionnaire, 2) the Charlson Comorbidity Index (CCI) to capture the weight of pre-existing comorbidities, 3) the Medical Outcomes Study Short-Form (SF-12) to assess general health including physical health (PCS) and mental health (MCS) subscales, and 4) the Self-Care Self-Efficacy Scale (SCSES) to measure the confidence of the participant in his/her self-care.

The Charlson Comorbidity Index (CCI) assigns differential weights to different comorbidities that reduce a patient's chance of survival. This instrument has been validated in different patient populations, including patients with COVID-19 and the reliability coefficient varies between 0.5 to 0.86 for predicting mortality based on comorbidities. RAND Medical Outcomes Survey Short Form 12 (SF-12) taps eight health concepts: physical functioning, bodily pain, role limitations due to physical health problems, role limitations due to personal or emotional problems, emotional well-being, social functioning, energy/fatigue, and general health perceptions, and a single item that indicates the perceived change in health. The reliability coefficient of SF-12 has been consistently above 0.7, demonstrating good reliability. Self-Care Self-Efficacy Survey (SCSES) was newly validated as a self-report measure for self-care self-efficacy among adults with chronic illness (mean age ranging from 65-77 years) and found high reliability with Cronbach's alpha coefficients ranging from 0.89-0.93. The COVID-19 Symptom Severity Scale (COVID-SRS) was developed for this study as an ordinal measure by adapting the CDC COVID-19 symptom checklist. The COVID-SRS instrument will let the patients rank their common COVID-19 symptoms into mild, moderate, or severe categories.

The average study completion time was 30 minutes. A total of 117 participants completed the study instruments. After accounting for omissions and errors in reporting, 87 out of 117 participants provided complete data on all measures.

Ethical considerations

This study was approved by the Institutional Review Board (IRB) at the University of Texas Medical Branch (UTMB) (approval no. 21-0259). The data collection process started with the potential participant signing the written informed consent electronically on an iPad and stored in the REDCap database. The participants were assured of the confidential nature of the study and their right to withdraw anytime from the study. All participants who chose to withdraw from the study were allowed to do so without penalty and the data collected so far were retained for reporting purposes. Extensive precautions were taken to ensure confidentiality and security of the electronic data including individualized passwords to prevent loss of data integrity. The data was de-identified assigning the participant an identification number by the REDCap survey system soon after the consent form was signed. The REDCap surveys were presented to the patient as an electronic survey and the data was collected electronically on a secured mobile device (iPad) connected to the secured network with a secured REDCap backend. All data were de-identified immediately after collecting them and only de-identified data were exported for analysis. Participants were encouraged to contact the principal investigator with their questions or concerns regarding this study.

Data analysis

Data collection and storage were accomplished using secured REDCap survey software. After the quality check, the de-identified data were exported and analyzed in aggregate using SAS software (SAS Institute, Cary, USA). A total of 120 participants completed the questionnaires; three records were removed due to participant duplication or withdrawal from the study. After cleaning data, the final sample included 117 participants. Instruments were scored using the steps in their published guidance instructions. For SF-12, any missing items resulted in a missing score for physical and mental component summary scores. For the SCSES, missing items greater than 50 percent resulted in a missing score. The missing score values for SF-12 were 14.5% and for SCSES were 1.7%. A total of 87 records were complete with the full set of data included in statistical analyses performed using the chi-square test, Fisher’s Exact, and Pearson correlations as shown in the results section.

## Results

This study was conducted among a purposive sample of COVID-19-positive, middle-to-older-age adults with pre-existing comorbidities. The sample characteristics are shown in Table 1.

**Table 1:** Selected Sample Characteristics

Demographics (n=117)	N	Percent
What is your gender?		
Male	58	52.3
Female	53	47.8
Age (approximate range in years)		
40-60 years	42	35.9
61-70 years	36	30.8
71-90 years	39	33.3
What is your marital status?		
Steady partner	82	71.9
Single	17	14.9
Widowed	15	13.2
How many children are there in your family?		
0	21	18.1
1-2	49	42.2
3-4	35	30.2
>4	11	9.5
How would you describe your race or ethnicity?		
African American/Black	12	10.3
Caucasian/white	83	70.9
Hispanic/Latino	17	14.5
Other	5	4.3
What is your highest level of education?		
< High School	11	9.5
High School diploma/Vocational/Technical certificate	26	22.4
2-4 years college	59	50.9
Master's/Doctorate education	20	17.2
What do you expect your combined family income to be, this year?		
<=$40,000	23	21.9
$40,001-$75,000	25	23.8
>$75,000	57	54.3
How often do you go to your medical provider?		
Monthly	14	11.97
Every 3-6 months	62	52.99
Yearly	20	17.09
Only when I have any health concerns	19	16.24
Rarely goes to the doctor	2	1.71
What type of living arrangement do you have?		
Independent living	78	67.24
Living with family	33	28.45
Assisted living/retirement facility	1	0.86
Other	4	3.45

The sample categories were more or less equivalent in terms of gender and age. The sample consisted of 52.3% men and 47.8 % women. Similarly, participants were approximately evenly distributed in age groups of less than 60 years, 61-70 years, and 71 and over of age. Most of the participants had some degree of family support with steady partners and children. However, the sample was less ethnically diverse with most of the participants being Caucasians/white (70.9%), with a smaller number of Hispanics (14.5%), African Americans/Black 10.3%, and only 4.3% of other ethnicities including Asians and Native Americans. Most people had a high school education.

We used the Charlson Comorbidity Index (CCI) modified in 2014 to measure the burden of comorbidities and it provided a list of comorbidities as well as a composite score denoting the cumulative burden of multiple comorbidities. Table 2 shows the presence of medical conditions reported by the participants through the Charlson Comorbidity Index.

**Table 2:** Distribution of comorbidities measured by the Charlson Comorbidity Index DM: diabetes mellitus; CVA: cerebrovascular accident

Medical Conditions (n=117)	Percent
Hypertension	42%
Diabetes	31%
Pulmonary disease/ asthma	19%
Depression	14%
Cancer (solid tumor)	11%
Myocardial infarction	8%
Congestive heart failure	8%
Peripheral vascular disease or bypass	6%
Renal disease	6%
Rheumatic or connective tissue disease.	5%
Mild liver disease	4%
Cerebrovascular disease or transient ischemic disease	3%
Lymphoma	2%
Diabetes with end-organ damage( If end-organ damage, do not check 'yes' to DM).	1%
Severe liver disease	1%
Gastric or peptic ulcer	1%
Hemiplegia (If Hemiplegia, do not check 'yes' to CVA).	0%
Leukemia	0%
A metastatic solid tumor (If Metastatic, do not count cancer separately).	0%
Dementia or Alzheimer's	0%
HIV or AIDS	0%
Skin ulcers/cellulitis	0%

The cardiometabolic disease spectrum including hypertension (42%) and diabetes without organ damage (31%) were the most prevalent comorbidities among the COVID-19-positive adults who participated in this study. The prevalence of pulmonary disease (19%) was notable, though CCI does not differentiate the pulmonary disease conditions. The prevalence of depression (14%) was noteworthy as a singular diagnosis. Solid tumors ranked fifth in the list, though CCI doesn’t differentiate the sites of the tumor. Older adults often carry a cluster of comorbidities that cumulatively impact a person’s health. The CCI score is considered a composite measure of the cumulative impact of the co-existing comorbidities, rather than the effect of a single comorbidity considered alone in a person.

**Figure 1:**
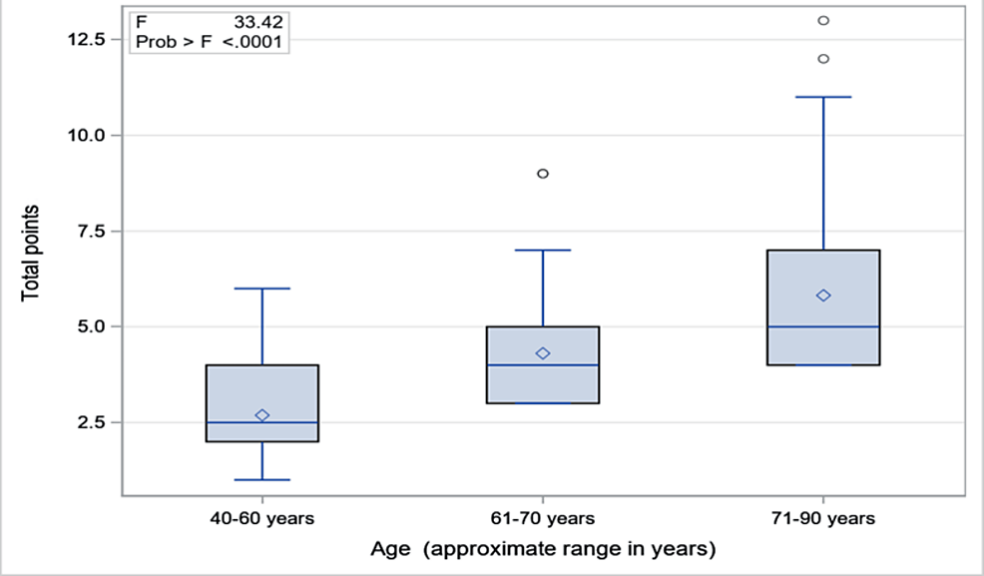
Charlson Comorbidity Index Score to Age: Box Whisker Plot

When considering the association between demographics and CCI score, age was the only statistically significant factor influencing the comorbidity burden of at-risk individuals who contracted COVID-19 (p<0.0001) As shown in Figure 1, there was a trend of an approximate doubling of the comorbidity burden from age 40-60 years (mean 3.2, SD 1.1) to age 71-90 years range (mean 6.9, SD 2.3).

The existing literature alludes that the COVID-19 symptoms are largely associated with the virulence of the COVID-19 viral strain and the patient’s vaccination status. However, we proposed that the patient's health determinants such as his/her pre-existing comorbidities, general health status, and self-care ability also may mediate symptom management and disease prognosis. This study was conducted during the third wave of COVID-19 caused by the relatively less virulent Omicron virus. In this study, we captured the perceived severity of the participant’s COVID-19 symptoms into mild, moderate, and severe categories using the COVID-19 symptom rating scale. The top five most severe symptoms reported were fatigue, body aches/muscle aches (myalgia), cough, congestion/runny nose, and sore throat. The rest of the typical COVID-19 symptoms such as fever, night sweats, loss of taste, loss of smell, nausea/vomiting, and abdominal pain/diarrhea were rated only mild to moderate by most participants as shown in Table 3. 

**Table 3:** Distribution of COVID-19 Symptoms Measured by the COVID-19 Symptoms Rating Scale

	N	Responses					
		None	Mild	Moderate	Severe		
		%	%	%	%	Mean	SD
Fever or Chills	113	22.1	38.1	29.2	10.6	2.3	0.9
Cough	117	5.1	27.4	48.7	18.8	2.8	0.8
Breathing difficulty	116	44.8	27.6	21.6	6.0	1.9	0.9
Fatigue	116	9.5	19.0	44.8	26.7	2.9	0.9
Muscle ache/ Body ache	116	17.2	25.9	34.5	22.4	2.6	1.0
Headache	116	21.6	32.8	31.0	14.7	2.4	1.0
Loss of smell	114	74.6	15.8	6.1	3.5	1.4	0.8
Loss of taste	113	71.7	17.7	7.1	3.5	1.4	0.8
Sore throat	117	17.9	31.6	32.5	17.9	2.5	1.0
Congestion/runny nose	117	6.8	24.8	50.4	17.9	2.8	0.8
Nausea or vomiting	116	68.1	19.8	10.3	1.7	1.5	0.8
Abdominal pain/diarrhea	117	62.4	24.8	9.4	3.4	1.5	0.8
Night sweats	115	47.8	22.6	23.5	6.1	1.9	1.0

We also explored the effects of the individual health determinants such as self-care self-efficacy (SCSES), general physical health status (SF-12 PCS), general mental health status (SF-12 MCS), and comorbidity burden (CCI) on the participants’ COVID-19 symptom severity as shown in the Table. 4. 

**Table 4:** Correlation of COVID-19 Symptoms to Comorbidities, General Health Status and Self-Care Self-Efficacy * Statistically significant value PCS: physical component summary; MCS: mental component summary

Pearson Correlation Coefficients, N = 87 Prob > |r| under H0: Rho=0				
	Overall Self-Care Self-Efficacy Score (SCSES)	Physical health T-score - SF12 (PCS)	Mental health T-score - SF12 (MCS)	Charlson Comorbidity Index Score (CCI)
Fever or Chills	0.15145 0.1614	-0.14191 0.1898	-0.26373 0.0136*	-0.11498 0.2889
Cough	0.06964 0.5216	-0.07869 0.4688	-0.24291 0.0234*	0.19616 0.0686
Breathing difficulty	0.03037 0.7801	-0.24856 0.0203*	-0.36236 0.0006*	0.00476 0.9651
Fatigue	-0.03101 0.7756	-0.27889 0.0089*	-0.40493 0.0001*	-0.00708 0.9481
Muscle ache/ Body ache	0.01453 0.8937	-0.32628 0.0020*	-0.40467 0.0001*	-0.05702 0.5999
Headache	-0.04176 0.7009	-0.31728 0.0028*	-0.36748 0.0005*	-0.17464 0.1057
Loss of smell	-0.14582 0.1778	-0.16267 0.1322	-0.12910 0.2334	-0.21289 0.0477*
Loss of taste	-0.14342 0.1851	-0.19577 0.0692	-0.19705 0.0673	-0.18626 0.0841
Sore throat	-0.11926 0.2712	-0.20623 0.0553	-0.16130 0.1356	-0.08878 0.4135
Congestion/runny nose	0.13451 0.2142	-0.15493 0.1519	-0.26433 0.0134*	0.06157 0.5711
Nausea or vomiting	-0.02538 0.8155	-0.21777 0.0427*	-0.33472 0.0015*	0.00828 0.9393
Abdominal pain/diarrhea	0.02485 0.8193	-0.21684 0.0437*	-0.22356 0.0374*	0.13569 0.2101
Night sweats	0.06930 0.5236	-0.30598 0.0039*	-0.25940 0.0153*	-0.15222 0.1593

The patient’s general health status (SF-12) was associated with the severity of many of the COVID-19 symptoms but a differential impact was evident when the physical health status subscale (SF-12 PCS) and mental health status subscale (SF-12 MCS) were considered separately. Interestingly, mental health status (MCS) showed a stronger association with most COVID-19 symptoms than physical health status (PCS). Fatigue and myalgia (body ache/muscle ache) showed a statistically significant correlation with mental health status (p<0.0001) followed by headache and breathing difficulties (p<0.001). Among the gastrointestinal symptoms, nausea/vomiting (p<0.01) and abdominal pain/diarrhea (p<0.05) also showed statistically significant association with mental health status. A less statically significant correlation was observed between mental health and febrile symptoms such as fever/chills, cough, congestion/runny nose, and night sweats (p<0.05).

The correlation between SF-12 PCS and COVID-19 symptoms of fatigue, myalgia, and headaches showed a less statistically significant correlation with physical health status (p<0.01) compared to that of mental health status. Night sweats (p<0.01), breathing difficulties, and gastrointestinal symptoms including nausea/vomiting, and abdominal pain/diarrhea (p<0.05) also showed statistically significant association with physical health status. Interestingly, ‘loss of smell’, which is the hallmark symptom of COVID-19, displayed a statistically significant correlation only with the Charlson Comorbidity Index (p<0.05), and it was associated neither with SF-12 nor with SCSES scores. It seems paradoxical that no COVID-19 symptoms other than ‘loss of smell’ reached statistical significance to the Charlson Comorbidity Index, though as described above most of these symptoms were associated with SF-12 scores.

The SF-12 MCS subscale demonstrated a statistically significant (p<0.01) correlation with the diagnosis of depression. This association of depression with the mental health subscale of SF-12 validates MCS as a true measure of mental health symptoms as used in this study. There was no statistically significant correlation between self-care self-efficacy scores and the COVID-19 symptoms indicating that symptom severity does not have any association to the person’s self-care ability.

## Discussion

This study explored the influence of selected patient characteristics including comorbidity burden, general health status, and self-care self-efficacy on COVID-19 symptoms among middle to older adults with COVID-19 treated in an outpatient setting. This study was conducted during the third wave of COVID-19 in which the Omicron variant was the predominant strain. Though the omicron strain was not as virulent as the previous COVID-19 variants, individuals with comorbidities were still vulnerable to complications and even death from COVID-19 illness. Most people were vaccinated with at least two doses of COVID-19 vaccines by the time the omicron outbreak began, resulting perhaps in milder symptoms and hence outpatient treatment became feasible. However, the risk assessment for severe infections during outpatient management of COVID-19 is still an under-researched area. The research literature is scarce on the influence of unique individual characteristics which might complicate recovery from COVID-19, while patients are self-managing their care in isolation within the community. The aging population often must deal with multiple chronic illnesses at the same time as COVID-19 symptom management and the cumulative impact of these illnesses often complicates recovery. We used the Charlson Comorbidity Index to explore the cumulative burden of the comorbidities among COVID-19-positive adults, and the results showed that advanced age significantly compounded the comorbidity burden among them. This finding concurs with previous study findings that older age and lower socioeconomic status make a person vulnerable to COVID-19 disease and its complications [26,27].

Through this study, we identified diabetes, hypertension, pulmonary diseases, solid tumor cancers, and depression as the top five most prevalent comorbidities among COVID-19-positive older adults who received COVID-19 monoclonal antibody infusion in outpatient settings. It is documented that cardiometabolic risk factors such as diabetes, hypertension, cardiovascular diseases, and obesity ranked high among the list of comorbidities leading to high mortality within the first two waves of COVID-19 disease [28]. Consequently, it led to the prioritization of hospitalization and lifesaving treatments such as monoclonal antibody infusion for older adults with cardiometabolic risk factors. However, findings from this study showed that not only the age and comorbidities but also the patient’s general health status influence the severity of the patient’s symptoms.

Notably, mental health status was more significantly related to the severity of COVID-19 symptoms than physical health, and this attests to the need to recognize poor mental health as a ‘hidden pandemic’ during the COVID-19 crisis, consistent with the increase in prescribing of medications for depression, anxiety and other mental health conditions during the COVID-19 pandemic [29]. This data shows that many patients suffer from covert psychological distress that either contributes to or aggravates their COVID-19 symptoms and it will not be evident unless we use a standardized scale such as SF-12 to measure their general health status. Also, The SF-MCS scores were correlated to a diagnosis of depression, validating it as a true measure of mental health symptoms. The findings from our study underscore the critical need for clinicians and patient care partners to give due attention to the patient’s general health status, specifically, mental health status and any mental health symptoms, into the equation of risk assessment, risk stratification, diagnosis, and management of COVID-19-positive adults with comorbidities. 

The findings from this study revealed that none other than the COVID-19 hallmark symptom of ‘loss of smell’ was associated with comorbidity burden, while all other COVID-19 symptoms were associated with the patient’s general health status. This finding calls for a closer look into the symptomatology of COVID-19 and its link to the inflammatory profile of patients with comorbidities for guidance in the development of possible clinical indicators and biomarkers of severe COVID-19 disease.

In this study, we found no significant relationship between the COVID-19 symptoms and self-care self-efficacy of the patients during their COVID-19 illness. However, a person’s self-care and self-efficacy are major determinants in the odds of survival during a pandemic, when isolation and quarantine can disrupt resources. Older adults are vulnerable to psychological distress and lack of access to care due to isolation and other COVID-related mitigation measures during a pandemic response. Thus, interventions to strengthen self-care and self-efficacy are of paramount importance during this time. More research is needed to develop and test age-appropriate interventions to strengthen self-care during pandemics, to mitigate loneliness and isolation, which are major causes of morbidity and premature death in the older population [30].

The limitations of the current study include a cross-sectional study design and convenient sampling methods. Also, data collection was done by self-reports, which has an inherent weakness of reporting bias. In this study, we assessed the impact of three selected intrapersonal variables: comorbidities, self-care self-efficacy, and general health status on COVID-19 symptoms. Though vaccination status is an important factor influencing the severity of COVID-19 symptoms, it was not a variable considered in this study. Within the boundaries of the above-mentioned study limitations, the findings from this study will be generalizable to individuals with COVID-19 infection and comorbidities, to strategize treatment for them in outpatient settings. Other strengths of our study include a balanced representation of men and women, the use of a comprehensive, reliable, and well-validated tool (SF-12) to assess the physical and mental health functions of our participants, assessment of self-care self-efficacy with a validated tool and our use of the COVID-19 Symptom Severity Scale (COVID-SRS) to capture a wide array of COVID symptoms.

## Conclusions

With the advent of more precise outpatient therapies to treat COVID-19, the new norm is to treat patients with COVID-19 in outpatient settings. The lack of screening resources to routinely evaluate clinically relevant risk factors (such as mental health and self-efficacy) in community settings is a major obstacle in identifying at-risk individuals and prioritizing interventions for them. The study's goal was to develop comprehensive, valid, reliable, and pragmatic criteria to capture the relevant risk factors of severe COVID-19 symptoms of middle-aged to older adults living in community-based isolation because of COVID-related mitigation measures and treated on an outpatient basis for COVID-19. This study's findings, such as the association of COVID-19 symptoms and patients’ health status, will potentially increase our understanding of individual health determinants such as comorbidities, self-care self-efficacy, and psychosocial/general health status as factors that influence the severity of COVID-19 infection. Also, the association of the COVID-19 symptom of loss of smell with the Charlson Comorbidity Index should be further explored with a rigorous study design in a sample of patients with long COVID because the loss of smell is a hallmark symptom of the ‘long COVID’ as well. Though self-care self-efficacy was not correlated to COVID-19 symptoms in this study, it is an important concept contributing to the well-being of older adults and needs to be explored with a more rigorous study design in future studies.

The findings from this study will provide evidence for rational recommendations for improving clinical outcomes in this population by promoting self-care behaviors and delivering strategies that can be integrated into COVID-19 disease management. The risk assessment tools used in this study can be integrated into electronic medical records to quickly make risk-level-specific clinical decisions during pandemics. Keeping COVID-19-positive older adults at home within the community has inherent benefits, including preventing nosocomial infections and preventing COVID-19 transmission, but it also might increase the risk of social isolation and loneliness, thus, a need to have a balance between public health and personal health. With more medications and other interventions becoming more available for outpatient treatment of COVID-19, community-based outpatient care will free up inpatient beds for patients with non-COVID-19 acute care needs. This study will help streamline efforts to tailor risk-level specific, cost-effective, and safe community-based infectious disease treatment protocols and enhance our disaster preparedness.
